# Everolimus and sirolimus in the treatment of cardiac rhabdomyomas in neonates

**DOI:** 10.1038/s41390-025-04043-8

**Published:** 2025-04-26

**Authors:** Daniel Hurtado-Sierra, Judy X. Ramos Garzón, Sandra L. Romero-Guevara, Angie Y. Serrano-García, Lyda Z. Rojas

**Affiliations:** 1 Pediatric Cardiology Unit, Instituto del Corazón de Bucaramanga, Bucaramanga, Colombia; 2 Nursing School, Universidad Industrial de Santander, Bucaramanga, Colombia; 3 Research Center, Fundación Cardiovascular de Colombia, Floridablanca, Colombia

## Abstract

**Background and objectives:**

Cardiac rhabdomyoma (CR) is the principal cardiac tumor diagnosed in pediatric age and is commonly associated with tuberous sclerosis complex. In some patients, these masses can cause heart failure and difficult-to-control arrhythmias. There are multiple case reports on use of mammalian target of rapamycin (mTOR) inhibitors, everolimus or sirolimus, in treatment of CRs. We reviewed the current data regarding effectiveness of everolimus and sirolimus in treating of CRs in newborns with hemodynamic repercussions.

**Methods:**

This systematic review was reported according to the PRISMA guidelines. The EBSCO, PubMed, EMBASE, and Lilacs databases were searched for full-text articles reporting the use of everolimus or sirolimus in the treatment of CRs in neonates and infants.

**Results:**

Thirty-one articles met inclusion criteria, totaling 48 patients. Hemodynamic instability prompted treatment in 89.5% of cases. Everolimus was used in 83.3% of cases and sirolimus in 16.6%. The median treatment duration was 67 days, with a 57 ± 23% average CR size reduction. Common adverse events included hypertriglyceridemia, infections, and hematological abnormalities.

**Conclusions:**

mTOR inhibitors appear effective and safe for treating CRs in neonates and infants. The average daily doses were 1.03 mg/m²/day for everolimus and 1.37 mg/m²/day for sirolimus. Randomized controlled clinical trials are necessary to confirm these findings and establish optimal treatment protocols.

**Impact:**

- Currently, there are no results from randomized clinical trials evaluating the efficacy of mammalian target of rapamycin inhibitors in patients with symptomatic cardiac rhabdomyomas.
- This is the first systematic review that evaluates the efficacy and safety of the use of everolimus and sirolimus in the non-surgical treatment of cardiac rhabdomyomas with hemodynamic repercussions in neonates.
- Everolimus and sirolimus may be particularly useful in the neonatal period when the hemodynamic complications caused by cardiac rhabdomyomas are more severe.

## Introduction

Cardiac rhabdomyoma (CR) is the principal cardiac tumor diagnosed in the pediatric age.^1^ Seventy-five percent of affected patients are under one year old, with an approximate incidence in newborns of 0.02 to 0.08%.^2^ Sixty to eighty percent of CR cases are associated with tuberous sclerosis complex (TSC), an autosomal dominant disorder that causes the growth of hamartomas in multiple organs, including the heart.^3^ Mutations present in TSC deactivate the genes encoding the hamartin (TSC1) or tuberin (TSC2) proteins, which are responsible for inhibiting the mammalian target of rapamycin (mTOR), a family of serine-threonine kinases involved in the regulation of cell growth and proliferation.^4^

Most CRs present as multiple masses located mainly in the ventricles.^1,2^ A significant number of CRs undergo spontaneous regression in early childhood. Although most patients may remain asymptomatic, in some cases, these masses can obstruct blood flow, cause heart failure, and difficult-to-control arrhythmias^.^^1,5–9^ Death in the fetal and early neonatal period can occur due to the obstructive effect of large masses or incessant arrhythmias leading to cardiogenic shock.^10–12^ Surgery is reserved for cases with severe hemodynamic compromise^5^; however, it is not without complications and risk of death.^13–17^

Although mTOR inhibitors (mTORi) such as everolimus and sirolimus have demonstrated their effectiveness in treating TSC-associated tumors such as subependymal giant cell astrocytoma (SEGA) and renal angiomyolipomas,^18,19^ there are currently no results from randomized clinical trials evaluating the efficacy of these drugs in patients with symptomatic CRs.^13,16,17^

There are multiple case reports in the literature on the use of everolimus or sirolimus as a therapeutic option (off-label) in the management of RC,^5,13,16,20^ with no consensus on essential aspects such as dose, duration of treatment, and adverse effects.^16^ This study aimed to review and synthesize the effectiveness of mTORi (everolimus and sirolimus) in treating of CRs in newborns with hemodynamic repercussions.

## Methods

This review was reported according to the guidelines of the PRISMA statement.^21^ This review was not registered in any publicly accessible database for systematic reviews.

### Search strategy and data sources

A literature search was conducted for articles published up to July 16, 2023, in four electronic databases (EBSCO, PubMed, EMBASE, and Lilacs) with no language restrictions. Initially, terms related to the PICO elements were used. Population and intervention-related terms were combined: Infant [MeSH], Newborn [MeSH] OR Neonate [MeSH], MTOR inhibitor [Free], OR Everolimus [Free] OR Sirolimus [Free], Rhabdomyoma [Free]. The search algorithms were: (((((Infant, Newborn[Title/Abstract]) OR (Neonate[Title/Abstract])) AND (MTOR inhibitor[Title/Abstract])) OR (Everolimus[Title/Abstract])) OR (Sirolimus[Title/Abstract])) AND (Rhabdomyoma[Title/Abstract]) and (newborn:ti,ab OR neonate:ti,ab) AND (everolimus:ti,ab OR sirolimus:ti,ab OR ‘mtor inhibitor’:ti,ab) AND rhabdomyoma:ti,ab and TX Infant, Newborn AND TX Everolimus OR TX Sirolimus TX Rhabdomyoma and Infant, Newborn OR Neonate AND MTOR inhibitor OR Everolimus OR Sirolimus AND Rhabdomyoma. The final searches were merged and managed for screening in the rayyan.ai web application.

### Eligibility criteria and study selection

Studies were eligible if: (1) the study population consisted of neonates and infants; (2) they used everolimus or sirolimus for the treatment of CR; (3) the outcome of interest was the size of the CR causing intractable cardiac arrhythmias or hemodynamic instability, and (4) any epidemiological design was included. Abstracts from scientific events and studies conducted in animals were excluded. Initially, four reviewers (two pairs) independently examined the titles and abstracts of all identified studies according to the selection criteria. Subsequently, full-text articles that met the selection criteria were retrieved and read entirely, and the eligibility criteria were reapplied. Any disagreements were resolved through consensus or consultation with the research team’s pediatric cardiologist and echocardiographer (DHS).

### Data extraction

A data extraction form was created to collect relevant information from the studies included in the Microsoft Excel program. Three reviewers independently extracted the following data from each study: first author’s name, year of publication, country, continent, study design, gestational age of the newborn, sex, weight, height, single or multiple CR, CR location, initial CR size (cm^2^ or mm), the reason for starting treatment (hemodynamic/arrhythmias), age at the start of treatment, mTORi used (everolimus/sirolimus), initial mTORi dose, cumulative mTORi dose, target serum level of mTORi, achieved serum mTORi level, changes made to the initial dose, reduction in CR size (mm, cm^2^, or %), hemodynamic improvement or resolution of arrhythmia, total treatment duration, adverse pharmacological effects, treatment suspension or dose reduction due to adverse effects, rebound after treatment suspension (mm, cm^2^, or %), treatment restart in case of rebound, duration of treatment in case of a second cycle, and total follow-up time (years). Any disagreements were resolved through consensus or consultation with a fourth independent reviewer. Finally, to clarify or complete relevant information, some of the authors of the publications were contacted via email.

### Quality assessment of studies

The methodological quality of the included studies was evaluated using the Joanna Briggs Institute (JBI) critical appraisal checklist for case reports.^22^ This evaluation was conducted independently by two reviewers, and in case of disagreement, it was discussed until a consensus was reached; otherwise, a third reviewer intervened. The checklist evaluated eight essential criteria and was standardized for this review as follows: (1) sociodemographic characteristics of the patient (report of gestational age at birth, sex, and weight of the newborn); (2) detailed timeline of the patient’s clinical history (report of CR dimensions and clinical condition at least two points in time); (3) current clinical condition (description of hemodynamic compromise and/or arrhythmia); (4) diagnostic methods and results (report of serum levels of mTORi and CR dimensions assessed by echocardiography); (5) description of the therapeutic procedure (detail of the dose and duration of mTORi treatment); (6) patient’s post-intervention status (report of CR size and evolution of the hemodynamic condition and/or arrhythmia); (7) identification of adverse events (specification of the occurrence or absence of side events) and 8) lessons learned (recommendations or implications for clinical practice).

### Statistical analysis

The data obtained were synthesized narratively, and descriptive statistics and frequency measurements were carried out (tables and supplementary material 1). The heterogeneity of the results did not allow meta-analysis.

## Results

### Selection of studies

**Fig. 1:**
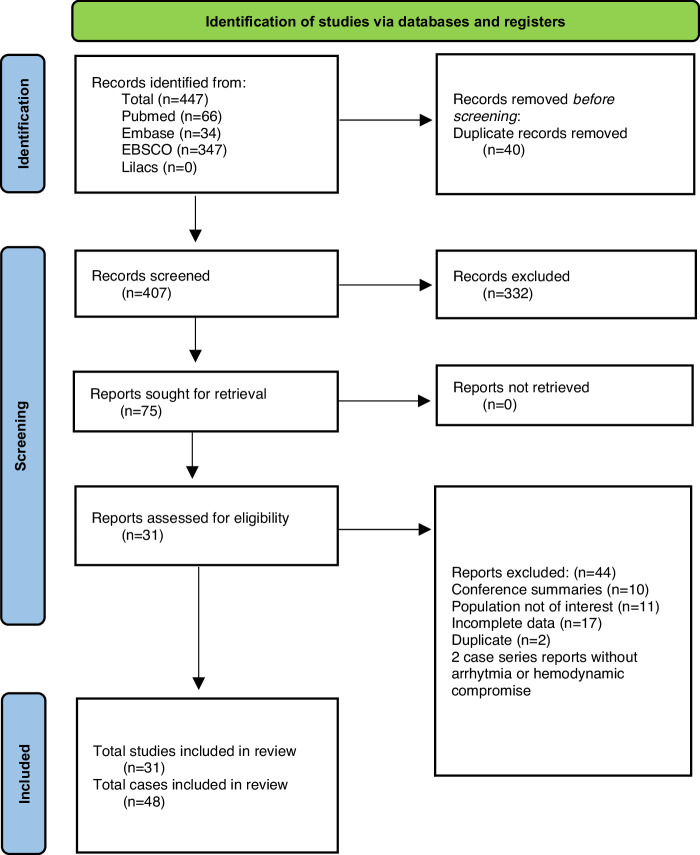
PRISMA flow diagram. Diagram detailing literature search and article screening.

The initial search identified 407 studies, of which 31 met the inclusion criteria, totaling 48 cases. Figure 1 shows the flow diagram of study selection and exclusion.

### Characteristics of the studies

The studies were published from 2012 to 2023, primarily reported from Asia (*n* = 26; 54.17%), followed by North America (*n* = 10; 20.83%), Europe (*n* = 6; 12.50%), Latin America (*n* = 5; 10.42%), and Oceania (*n* = 1; 2.08%). Case reports constituted 58.33% (*n* = 28) of the studies, followed by case series (*n* = 15; 31.25%) and case-control studies (*n* = 5; 10.42%).

Among the patients, 53.8% were full-term newborns, and 70.4% were male, with a median weight of 2927 grams (Q1 = 2150; Q3 = 3240). In 72.9% of cases, CR was multiple (more than one), primarily located in the left ventricular outflow tract (36.1%), followed by the right ventricular cavity (27.6%). Hemodynamic instability was the main reason for initiating treatment with a mTORi (89.5%). Everolimus was administered in 83.3% of cases (*n* = 40) and sirolimus in 16.6% (*n* = 8), with a median age of drug initiation of 6 days (Q1 = 3; Q3 = 18). Other study and population characteristics can be found in Tables 1 and 2.

**Table 1:** Grouped Characteristics of Included Studies and Cases.

Studies/Cases	*n* (%)
**Gestational age of the NB (weeks), median, Q1-Q3, (n** = **36)**	36.5 (36; 38)
**Full-term NB (n** = **39), yes**	21 (53.85)
**Sex of the NB (n** = **44), male**	31 (70.45)
**Weight (g), median, Q1-Q3, (n** = **40)**	2927 (2150; 3240)
**Rhabdomyoma (n** = **48)**	
Multiple	35 (72.92)
Single	23 (27.08)
**Location of the rhabdomyoma (n** = **47)**	
LVOT	17 (36.17)
RV	13 (27.66)
LV	10 (21.28)
RVOT	4 (8.51)
RV/LV	2 (4.26)
RA	1 (2.13)
**Reason for initiating treatment (n** = **48)**	
Hemodynamic	43 (89.58)
Hemodynamic/Arrhythmia	3 (6.25)
Arrhythmia	2 (4.17)

*LV* left ventricle, *LVOT* left ventricular outflow tract, *NB* newborn, *RA* right atrium, *RV* right ventricle, *RVOT* right ventricular outflow tract.

**Table 2:** Individual characteristics of the included cases (*n* = 48).

Author/Publication years	Country	Study Design	Full-term pregnancy	Sex	Weight (g)	Rhabdomyoma	Rhabdomyoma Location	Initial size (mm)	Reason for treatment initiation
Demir et al.^38^	Turkey	CR	Yes	Male	3400	Multiple	RVOT	25	Hemodynamic
Breathnach et al.^39^	Ireland	CR	Yes	Male	–	Multiple	LVOT	15 × 12	Hemodynamic
Goyer et al.^40^	Canada	CC	No	Female	980	Multiple	RV	16 × 11	Hemodynamic
Goyer et al.^40^	Canada	CC	No	Female	1670	Multiple	LVOT	27.2	Hemodynamic
Wagner et al.^41^	Germany	CR	Yes	–	2955	Multiple	LV	37 × 21 x 21	Hemodynamic
Aw et al. 2016^12^	Canada	CC	No	Female	980	Multiple	RV	14.2 × 12	Hemodynamic
Aw et al. 2016^12^	Canada	CC	No	Female	1670	Multiple	LVOT	27.2 × 6.5	Hemodynamic
Aw et al. 2016^12^	Canada	CC	Yes	Male	3300	Multiple	LVOT	8 × 7	Hemodynamic
Colaneri et al.^42^	Italy	CR	No	Male	2000	Multiple	LV	40 x 35 x 40	Hemodynamic
Chang et al.^43^	Taiwan	CS	Yes	Male	2880	Single	LV	–	Hemodynamic
Chang et al.^43^	Taiwan	CS	Yes	Female	2800	Multiple	LV	26.4	Hemodynamic
Chang et al.^43^	Taiwan	CS	Yes	Male	2720	Single	LVOT	30 × 20	Hemodynamic
Lee at al.^44^	South Korea	CR	No	Male	1170	Multiple	LVOT	5.2 × 3.6	Hemodynamic
Schmidt-Fittschen et al. ^45^	Germany	CR	Yes	Male	3280	Single	RV	35 x 28 x 24	Hemodynamic
Martínez-García et al.^46^	Mexico	CR	Yes	Female	3040	Multiple	LV	47 × 40	Hemodynamic
Weiland et al.^47^	United States	CR	–	–	–	Multiple	RV	33 x 25 x 25	Hemodynamic
Weiland et al.^47^	United States	CR	–	–	–	Multiple	LVOT	10.6 × 9.6 × 9.7	Hemodynamic
Dhulipudi et al.^48^	India	CS	–	Male	3200	Multiple	RV	28	Hemodynamic
Dhulipudi et al.^48^	India	CS	–	Male	3200	Multiple	RV	36	Hemodynamic
Dhulipudi et al.^48^	India	CS	–	Male	4500	Single	LVOT	12	Hemodynamic
Dhulipudi et al.^48^	India	CS	–	Male	2800	Single	RVOT	24	Hemodynamic
Dhulipudi et al.^48^	India	CS	–	Male	2200	Single	RA	13	Hemodynamic
Garg et al. 2019^14^	United States	CR	Yes	Male	–	Multiple	RV/LV	40 × 37	Hemodynamic/Arrhythmia
Lawley et al.^49^	Australia	CR	No	Female	2250	Multiple	LVOT	–	Hemodynamic
Shibata et al.^50^	Japan	CR	Yes	Male	2029	Multiple	RV	35 × 21	Hemodynamic
Esmer - Sánchez et al.^51^	Mexico	CR	Yes	Male	–	Single	LV	30 × 22	Hemodynamic
Prasad et al.^52^	India	CR	No	Female	2100	Multiple	LV	41 × 31	Hemodynamic
Çetin et al.^53^	Turkey	RC	–	Male	7000	Multiple	LVOT	7.9 × 6.1	Hemodynamic
Nespoli et al.^7^	Italy	CR	Yes	Male	4660	Multiple	LVOT	10 × 10	Hemodynamic
Nir-David et al.^54^	Israel	CR	Yes	Female	3090	Single	RVOT	18	Hemodynamic
Relan et al.^55^	India	CR	Yes	Male	3025	Multiple	LV	45 × 35	Hemodynamic
Silva-Sánchez et al.^56^	Colombia	CR	Yes	Male	3170	Multiple	RV/LV	42 × 27	Hemodynamic/Arrhythmia
Tsuchihashi et al.^57^	Japan	CR	No	Male	1350	Multiple	RV	17.1 × 13.1	Hemodynamic
Beyazal et al.^58^	Turkey	CR	Yes	–	3700	Multiple	LVOT	7 × 6	Hemodynamic
Çetiner et al.^59^	Turkey	CR	Yes	Male	–	Single	LVOT	18 × 17	Hemodynamic
Inoue et al.^60^	Japan	CR	No	Male	–	Multiple	LVOT	10.7 × 7	Hemodynamic
Sagiv et al.^61^	United States	CR	Yes	Male	3800	Multiple	RV	–	Arrhythmia
Winkie et al.^62^	United States	CR	–	Male	–	Multiple	LVOT	10.3 × 7.5	Hemodynamic
Babaoğlu et al.^63^	Turkey	CS	–	Female	1950	Multiple	LV	36 × 33	Hemodynamic
Babaoğlu et al.^63^	Turkey	CS	–	Male	3200	Single	LVOT	19 × 15	Hemodynamic
Babaoğlu et al.^63^	Turkey	CS	–	Male	3300	Multiple	LVOT	27 × 21	Hemodynamic
Babaoğlu et al.^63^	Turkey	CS	–	Female	5500	Multiple	RVOT	13.2 × 10.7	Hemodynamic
Babaoğlu et al.^63^	Turkey	CS	–	Male	2975	Multiple	–	28 × 14	Hemodynamic
Babaoğlu et al.^63^	Turkey	CS	–	Male	2900	Multiple	LV	8 × 10	Arrhythmia
Babaoğlu et al.^63^	Turkey	CS	–	Male	2800	Single	RV	30 × 25	Hemodynamic/Arrhythmia
Hurtado-Sierra D et al.^64^	Colombia	CR	Yes	Female	2890	Single	RV	37 × 20.7	Hemodynamic
Hurtado-Sierra D et al.^64^	Colombia	CR	No	Male	2240	Multiple	RV	30 × 17.3	Hemodynamic
Montaguti et al.^65^	Italy	CR	Yes	Female	2980	Single	RV	42 × 28	Hemodynamic

*CC* Case-Control, *CR* Case Report, *CS* Case Series, *LV* Left Ventricle, *LVOT* Left Ventricular Outflow Tract, *RA* Right Atrium, *RV* Right Ventricle, *RVOT* Right Ventricular Outflow Tract.

### Doses of mTOR inhibitors and duration of treatment

Considering the heterogeneity in dose reporting, the information was consolidated into cumulative weekly dose in mg/m^2^ and mg/kg. The cumulative dose for everolimus in mg/m^2^ was calculated in 25 cases, with a median of 4.5 mg/m^2^/week (Q1 = 3.9; Q3 = 5), a minimum of 0.6, and a maximum of 31.5 mg/m^2^/week. The cumulative dose in mg/kg was estimated in 28 cases, with a median of 0.33 mg/kg/week (Q1 = 0.23; Q3 = 0.64), a minimum of 0.12, and a maximum of 1.47 mg/kg/week. The cumulative dose for sirolimus in mg/m^2^ was calculated in 5 cases, with a median of 10.5 mg/m^2^/week (Q1 = 8; Q3 = 10.9), a minimum of 4.2, and a maximum of 15.9 mg/m^2^/week. The cumulative dose in mg/kg was estimated in 6 cases, with a median of 0.74 mg/kg/week (Q1 = 0.70; Q3 = 1.4), a minimum of 0.42, and a maximum of 1.46 mg/kg/week (Table 3).

**Table 3:** Medication, doses, duration, and other characteristics of treatment with mTORi.

Studies/Cases	Median (Q1–Q3)
**mTORi**	
Everolimus, *n* (%)	40 (83.33)
Sirolimus, *n* (%)	8 (16.67)
**Age at treatment initiation in days (n** = **43)**	6 (3; 18)
**Cumulative dose of everolimus mg/m**^**2**^**/week (n** = **25)**	4.5 (3.9; 5)
**Cumulative dose of sirolimus mg/m**^**2**^**/week (n** = **5)**	10.5 (8; 10.9)
**Cumulative dose of everolimus mg/kg/week (n** = **28)**	0.33 (0.23, 0.64)
**Cumulative dose of sirolimus mg/kg/week (n** = **6)**	0.74 (0.70, 1.4)
**Maximum serum level of everolimus ng/ml (n** = **28)**	11.5 (8.7; 18)
**Maximum serum level of sirolimus ng/ml (n** = **6)**	26.5 (24.3; 42.1)
**Day maximum serum level reached (n** = **26)**	7.5 (6; 14)
**Changes in initial dose (n** = **48), yes**	20 (41.67)
**Duration of treatment in days (n** = **42)**	67 (36; 112)
**Presence of adverse events (n** = **48), yes**	20 (41.67%)
**Discontinuation of treatment due to AE (n** = **33), yes**	12 (36.36%)
**Rebound after discontinuing treatment (n** = **34), yes**	20 (58.82%)
**Final reduction in mass (%), mean (SD)**	57 ± 23
**Total follow-up time, years (n** = **35)**	0.83 (0.5; 1.7)
**Total follow-up time, months (n** = **35)**	10 (6; 21)

*AE* adverse event, *mTORi* mTOR inhibitor, *SD* standard deviation.

A high percentage of studies (68.4%) used the target serum level of mTORi reported in the EXIST studies of 5–15 ng/mL.^18,19^ The maximum serum level of everolimus was reported in 28 cases, with a median of 11.5 ng/mL (Q1 = 8.7; Q3 = 18), and for sirolimus, it was reported in 6 cases, with a median of 26.5 ng/mL (Q1 = 24.3; Q3 = 42.1). In more than 50% of the cases, the maximum serum level of mTORi was reached within 7 days of treatment. The median duration of treatment was 67 days (Q1 = 36; Q3 = 112). In 41.67% of cases, changes in the initial dose were reported, the main cause being serum levels of mTORi outside the established range (68.4%) and, to a lesser extent, adverse events (26.3%) (Table 4).

**Table 4:** Characteristics of mTORi administration in the included cases (*n* = 48).

Authors/ Year of Publication	Age at the Start of Tx (days)	Medication	Approx. Accumulated Dose mg/m^2^/week	Approx. Accumulated Dose mg/kg/week	mTORi Target Serum Level ng/mL	Max. Serum Level mTORi ng/mL	Initial dose changes	Duration of Tx (days)	AE	Tx Suspension Due to AE	Rebound	Final Mass Reduction (%)	Total Follow-up Time (months)
Demir et al.^38^	–	Everolimus	–	0.59	5 a 15	83.5 (day 7)	Yes	70	Yes	Yes	Yes	–	–
Breathnach et al. ^39^	10	Sirolimus	–	–	20	26 (day 7)	Yes	24	No	No	Yes	66	7.92
Goyer et al.^40^	20	Everolimus	–	0.71	5 a 15	13.7 (day 4)	No	34	No	Yes	No	–	10.68
Goyer et al.^40^	4	Everolimus	–	0.42	5 a 15	11.5 (day 15)	No	77	Yes	No	Yes	55.9	8.76
Wagner et al. ^41^	2	Everolimus	12.25	1.02	5 a 15	108 (day 4)	Yes	19	Yes	No	No	24.3	5.04
Aw et al. 2016^12^	20	Everolimus	7	0.71	5 a 15	13.7 (day 4)	No	34	No	Yes	Yes		21
Aw et al. 2016^12^	4	Everolimus	5	0.42	5 a 15	11 (day 6)	No	46	No	No	Yes	50	21
Aw et al. 2016^12^	1	Everolimus	3.3	0.21	5 a 15	10.2 (day 10)	No	36	No	No	Yes	50	18.84
Colaneri et al.^42^	7	Everolimus	10.5	0.77	5 a 15	16 (day 40)	Yes	70	No	No	No	80	9
Chang et al.^43^	7	Everolimus	2.17	0.14	3 a 7	5.59 (day 35)	Yes	187	Yes	No	Yes	–	–
Chang et al.^43^	5	Everolimus	2.31	0.15	3 a 7	20 (day 50)	Yes	94	No	Yes	Yes	86.7	23.04
Chang et al.^43^	2	Everolimus	4.55	0.32	3 a 7	16 (day 5)	Yes	90	No	Yes	Yes	80.5	9.96
Lee at al. ^44^	18	Sirolimus	15.9	1.46	10 a 20	42.1 (day 14)	Yes	57	No	No	No	55.7	9.6
Schmidt-Fittschen et al.^45^	11	Everolimus	–	0.33	6 a 10	–	No	319	Yes	No	Yes	77.1	24
Martínez-García et al.^46^	36	Everolimus	–	0.33	–	–	No	64	No	No	No	–	3.96
Weiland et al. ^47^	–	Sirolimus	–	0.7	5 a 15	–	No	30	No	No	Yes	86	9.96
Weiland et al. ^47^	–	Sirolimus	–	1.4	5 a 15	24.3 (day 12)	Yes	–	No	–	No	43.4	–
Dhulipudi et al.^48^	3	Everolimus	4.5	–	–	–	No	112	No	No	–	14.3	–
Dhulipudi et al.^48^	1	Everolimus	4.5	–	–	–	No	56	Yes	No	–	27.7	–
Dhulipudi et al.^48^	90	Everolimus	4.5	–	–	–	No	56	Yes	No	Yes	50	–
Dhulipudi et al.^48^	7	Everolimus	4.5	–	–	–	Yes	42	No	Yes	Yes	50	–
Dhulipudi et al.^48^	6	Everolimus	4.5	–	–	–	No	224	No	No	No	38.5	–
Garg et al. 2019^14^	4	Everolimus	2.1	–	–	–	Yes	–	Yes	–	–	–	6
Lawley et al.^49^	3	Sirolimus	10.93	0.79	5 a 15	69.7 (day 8)	Yes	60	No	Yes	Yes	–	12
Shibata et al.^50^	4	Everolimus	19.6	1.38	5 a 15	76.1 (day 3)	Yes	6	No	Yes	No	31.4	1.8
Esmer - Sánchez et al.^51^	4	Everolimus	31.5	–	5 a 10	26.8 (day 14)	Yes	730	Yes	No	No	73.3	24
Prasad et al.^52^	2	Everolimus	4.5	–	–	–	No	112	Yes	–	–	–	2.4
Çetin et al. ^53^	90	Everolimus	4.5	–	–	–	No	120	No	No	No	65.8	–
Nespoli et al.^7^	26	Everolimus	–	0.15	3 a 8	4.5 (day 5)	No	180	No	–	No	50	–
Nir-David et al. ^54^	120	Sirolimus	8	0.42	5 a 15	16.67 (day 6)	Yes	30	No	–	Yes	63	–
Relan et al.^55^	2	Sirolimus	10.5	0.7	10 a 15	27.1 (day 24)	Yes	344	No	No	Yes	90	37.2
Silva-Sánchez et al. ^56^	3	Everolimus	–	0.7	5 a 8	–	No	42	No	–	No	45.2	12
Tsuchihashi et al. ^57^	28	Everolimus	4.9	0.52	5 a 15	14.3 (day 14)	No	180	No	–	–	73	6
Beyazal et al.^58^	–	Everolimus	4.55	–	5 a 15	8 (day 8)	No	119	No	–	No	100	3
Çetiner et al. ^59^	7	Everolimus	0.6	–	–	–	Yes	–	Yes	–	–	86.1	24
Inoue et al.^60^	7	Everolimus	14	–	5 a 15	67 (day 7)	Yes	–	No	Yes	–	28	–
Sagiv et al. ^61^	8	Everolimus	–	0.23	5 a 10	11,4	No	35	Yes	Yes	Yes	75	4.92
Winkie et al.^62^	–	Sirolimus	4.2	–	5 a 15	–	No	–	Yes	–	–	70.8	10.92
Babaoğlu et al.^63^	2	Everolimus	–	1.47	5 a 15	37.1	No	25	Yes	No	–	3	–
Babaoğlu et al.^63^	4	Everolimus	–	0.31	5 a 15	9.3	No	70	Yes	–	–	64	36
Babaoğlu et al.^63^	3	Everolimus	–	0.33	5 a 15	11.3	Yes	70	Yes	Yes	No	46	6.96
Babaoğlu et al.^63^	105	Everolimus	–	0.12	5 a 15	12.6	Yes	56	No	Yes	Yes	66	6
Babaoğlu et al.^63^	6	Everolimus	–	0.24	5 a 15	7.6	No	84	No	–	–	42	24.96
Babaoğlu et al.^63^	8	Everolimus	–	0.34	5 a 15	8.9	No	42	No	–	–	36	9.96
Babaoğlu et al.^63^	6	Everolimus	–	0.35	5 a 15	8.5	No	35	–	–	–	48	6
Hurtado-Sierra D et al. ^64^	2	Everolimus	4.4	0.31	5 a 15	8.4 (day 6)	No	84	Yes	No	Yes	79.1	22.8
Hurtado-Sierra D et al. ^64^	3	Everolimus	3.9	0.24	5 a 15	11,5 (day 7)	No	121	Yes	No	Yes	78.6	15.6
Montaguti et al. ^65^	60	Everolimus	2.4	0.14	3 a 8	2.4 (day 15)	No	–	No	–	–	30.9	6

*AE* adverse event, *mTORi* mTOR inhibitor, *Tx* treatment.

### Effect of mTORi on CR size and long-term follow-up

In all cases, an improvement or resolution of the symptoms for which the treatment was initiated was reported. The average total reduction in CR size was 57 ± 23%. In 5 patients, an average CR reduction of 48.5 ± 33.8% was achieved between days 16 and 30 of treatment, and in 11 patients, the reduction was 50.3 ± 12.8% between days 31 and 60. The median total follow-up time was 10 months (Q1 = 6; Q3 = 21). In 34 cases, the evolution of CR after mTORi discontinuation was reported, finding an increase in the mass size (rebound) in 58.82% (20 patients). Treatment was restarted in 50% (10 out of 20 patients).

The association between the total percentage reduction of the mass and the maximum serum level of the mTORi was explored through linear regression, finding a significant relationship. For each unit ng/mL of mTORi, a reduction of 0.41% in the mass was observed (β = −0.41; 95% CI −0.75 to -0.08; *p* = 0.018). This association remained significant after adjusting for the medication and the newborn’s gestational age (*β* = −0.43; 95% CI −0.78 to −0.07; *p* = 0.020).

### Adverse events related to mTORi

Adverse events were reported in 41.6% of the cases (*n* = 20); however, only 6 patients (12.5%) required permanent treatment discontinuation. For the remaining cases, the dose was reduced, or the mTORi was temporarily suspended. The most common adverse events were hypertriglyceridemia, infections, and hematological abnormalities (Table 5).

**Table 5:** Main reported adverse events.

Author/year	mTORi	Adverse event	Maximum serum level reached ng/ml”	Permanent discontinuation of mTOR inhibitor
Demir et al.^38^	Everolimus	Lymphopenia, hypertriglyceridemia, diarrhea	83.5	No
Breathnach et al.^39^	Sirolimus	Hypertriglyceridemia	26	No
Goyer et al.^40^	Everolimus	Suspected infection	13.7	Yes
Aw et al. 2016^12^	Everolimus	Suspected infection	13.7	Yes
Colaneri et al.^42^	Everolimus	Hypertriglyceridemia, mucositis	16	No
Chang et al.^43^	Everolimus	Pneumonia, low weight/height ratio	20	Yes
Chang et al.^43^	Everolimus	Chickenpox, low weight/height ratio	16	Yes
Dhulipudi et al.^48^	Everolimus	Chickenpox	–	No
Lawley et al.^49^	Sirolimus	Neutropenia	69.7	No
Shibata et al.^50^	Everolimus	Coagulopathy (pulmonary hemorrhage), elevated liver enzymes, acne	76.1	Yes
Esmer - Sánchez et al.^51^	Everolimus	Hypertriglyceridemia	26.8	No
Relan et al.^55^	Sirolimus	Hypertriglyceridemia	27.1	No
Inoue et al.^60^	Everolimus	Leukopenia, acute kidney injury	67	No
Sagiv et al.^61^	Everolimus	Neutropenia	11.4	Yes
Babaoğlu et al.^63^	Everolimus	Pneumonia	37.1	No
Babaoğlu et al.^63^	Everolimus	Hypertriglyceridemia	12.6	No
Babaoğlu et al.^63^	Everolimus	Hypertriglyceridemia	11.3	No
Hurtado-Sierra D et al.^64^	Everolimus	Anemia, hypercholesterolemia	11.5	No
Hurtado-Sierra D et al.^64^	Everolimus	Anemia, hypercholesterolemia	8.4	No
Montaguti et al.^65^	Everolimus	Hypertriglyceridemia	2.4	No

*mTORi* mTOR inhibitor.

### Evaluation of study quality

**Fig. 2:**
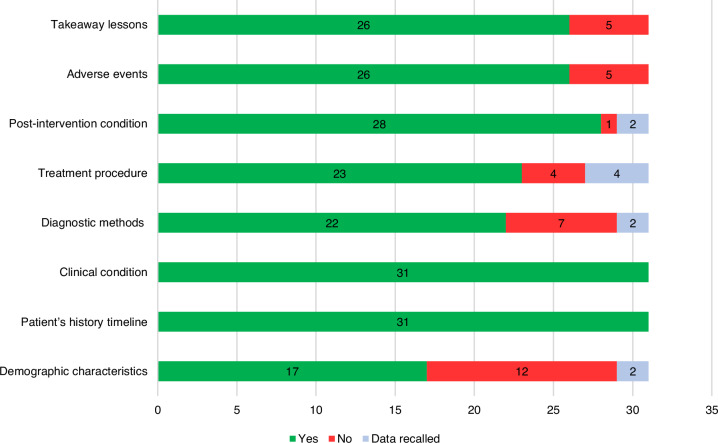
Quality assessment of included studies. Proportion of studies meeting each of the eight items on the Joanna Briggs Institute (JBI) Critical Appraisal Checklist for Case Reports. Green bars represent Yes (criterion met), red bars represent No (criterion not met), and light blue bars represent Data obtained from authors.

The detailed description of each article’s JBI Critical Appraisal Checklist can be reviewed in the Supplemental Table S1. Overall, the studies assessed mostly met the criteria outlined in the checklist. The criterion that showed the lowest compliance was demographic characteristics, with no data in 14 articles. Additionally, 9 articles did not meet diagnostic methods and outcomes criteria due to inaccuracies in CR dimensions or reported mTORi serum levels. With the collaboration of the articles’ authors, missing data were clarified for only two patients. Figure 2 provides a summary of the compliance percentage for each evaluated criterion.

## Discussion

There are randomized controlled clinical trials demonstrating the efficacy and safety of mTORi in the treatment of certain manifestations associated with TSC, such as subependymal giant cell astrocytoma, drug-resistant focal seizures, and renal angiomyolipomas in children.^18,19,23^ To date, there is one phase II clinical trial evaluating the efficacy of mTOR inhibitors in the treatment of symptomatic cardiac rhabdomyomas in children, but the results are not yet available.^13^

Our review revealed a significant increase in case reports and case series documenting the off-label use of mTORi in treating hemodynamically significant CRs. This increase has been particularly notable in the last five years, with more than 60% of case reports made in this period. The proven efficacy of mTORi in treating other TSC manifestations has led to the increasingly frequent use of these medications in symptomatic CRs.^16^

Although spontaneous regression of CRs is common and most patients remain asymptomatic, neonates are particularly vulnerable to hemodynamic complications or arrhythmias caused by these masses, which can lead to death in the early neonatal period.^1,5,9–11^ In our review, initiating treatment with mTORi was mainly a life-saving measure, such as in patients with severe ventricular outflow tract obstruction, interfering with ventricular contraction by giant masses, or incessant arrhythmias that were difficult to control.

There is significant variability in the age at which mTORi treatment is initiated, largely determined by the severity of hemodynamic compromise. In most cases reviewed, treatment was initiated within the first 7 days of life. Currently, no studies standardize the age at which mTORi treatment should commence in neonates with symptomatic CRs.^24^

There is no consensus on the doses of mTORi used for treating hemodynamically significant CRs. Fixed doses were used in some cases, while others calculated doses per kilogram or body surface area. We calculated the cumulative weekly dose based on information obtained from each report, observing wide variability for both everolimus and sirolimus. The accumulated dose of everolimus ranged widely from 0.6 to 31.5 mg/m^2^/week, primarily administered as a daily dose over 7 days. The average daily dose of everolimus was 1.03 mg/m^2^/day, significantly lower than the FDA-approved dose for treating SEGA and pharmacoresistant focal onset seizures associated with TSC (4.5 mg/m^2^/day).^18,23^ The accumulated dose of sirolimus ranged of 4.2 to 15.9 mg/m^2^/week, mainly administered daily over 7 days. The average daily dose of sirolimus was 1.37 mg/m^2^/day. Like us, Sugalska et al. could not determine a standardized dose of mTORi due to marked variability in the reported data.^16^

In 2017, Mizuno et al. reported the results of a pharmacokinetic study for precise dosing of sirolimus in pediatric patients with vascular anomalies, concluding that younger children, such as infants and neonates, require lower doses of sirolimus to achieve the target serum level, as well as having lower drug clearance compared to children older than 2 years.^25^ In this study, the average dose of sirolimus needed to achieve the target serum level close to 10 ng/ml was 1.8 mg/m^2^/day for children older than 2 years and ranged from 0.7 to 1.6 mg/m^2^/day for patients aged 3 weeks to 2 years. Based on available evidence and data from our review, we suggest that it is reasonable to use lower doses of mTORi in newborns and infants than those approved for other TSC-associated manifestations, changing doses according to the target serum level.

Recently, Ng et al. proposed a dosing and monitoring strategy for sirolimus in neonates and infants.^26^ For premature newborns, the proposed initial dose is 0.25 mg/m^2^/day; for term newborns - 0.5 mg/m^2^/day; for infants aged 2 to 6 months - 0.5 to 1 mg/m^2^/day; and for infants aged 6 months to 12 months - 1 to 1.5 mg/m^2^/day, with a 10 to 15% dose increase in all cases based on sirolimus serum level.^26^

Neonates and infants are predisposed to achieve high serum levels of mTORi due to the low activity of enzymes that metabolize these drugs.^26^ Everolimus and sirolimus are mainly metabolized through the CYP3A enzyme family, which is expressed at very low levels during gestation and remains functionally immature after birth, reaching only 30-40% of adult values at 4 weeks and full levels around 3 years.^27–29^

Although we found a significant relationship between the total percentage reduction in mass and mTORi serum level, the goal is to maintain the drug’s serum level within the reference range. In our review, the median maximum serum level for everolimus was within the reference range; however, some patients reported values as high as 108 ng/mL or 83.5 ng/mL. We also observed that the maximum mTORi level was reached early in the first week of treatment. Considering these data and the neonatal predisposition to achieve high mTORi serum levels, the first serum measurement of the drug should be performed in the first week after starting treatment. Thus, Ng et al. propose monitoring mTORi serum levels on days 3 and 7 after the first dose, continuing with weekly measurements until a stable state is achieved.^26^

Sirolimus and everolimus have been used interchangeably in preclinical studies; however, few studies have directly compared the two drugs.^30^ In the absence of comparative clinical trials, the specific selection of everolimus or sirolimus in treating manifestations associated with TSC will be guided by the best evidence published to date^.^^30^ For example, there is greater availability of controlled randomized clinical trials on the use of everolimus in treating SEGA, renal angiomyolipomas, and refractory epilepsy,^8,19,23^ or more controlled randomized clinical trials on treating lymphangioleiomyomatosis with sirolimus.^31^ For treating CRs with mTORi, only one double-blind, multicenter, placebo-controlled randomized clinical trial of everolimus in patients with TSC and symptomatic CRs (ORACLE) is currently underway.^13^

In a small case-control study, Aw et al. reported a reduction in CR size of at least 50% in the group of patients treated with everolimus over a period of 1.13 ± 0.33 months (median 29.5 days), compared to the 72.9 ± 53.03 months it took for the control group. The CR reduction rate for the treatment group was 11.8 times faster than for the control group.^12^ In our review, all included cases reported a significant reduction in CR size and hemodynamic improvement and/or resolution of arrhythmia that prompted the initiation of treatment. Some patients experienced a reduced CR size of nearly 50% when nearing completion of the first or second month of treatment. While the types of studies included in this review do not allow for generalization of CR response to mTORi treatment, it is evident that these medications are particularly useful in the neonatal period when hemodynamic complications from these masses are more critical.

The optimal duration of mTORi treatment for hemodynamically significant CRs is not established. Cleary and McMahon reported significant variation in treatment duration among different centers, ranging from approximately 1 to 3 months.^26^ Our review reported treatment duration in 87.5% of cases, with a median of 2.2 months. In most patients, mTORi was discontinued once a significant reduction in CR size was achieved and the initial hemodynamic condition that prompted medication initiation was surpassed. In a smaller proportion, mTORi was discontinued due to adverse events. Sugalska et al. could not correlate treatment duration and CR reduction due to divergence found in reported data.^16^

Although not all authors in our review reported the evolution of CRs once treatment was discontinued, rebound growth of the mass occurred in 20 cases, becoming significant enough in some of them to necessitate restarting mTORi. In Sugalska et al.‘s review, the post-treatment evolution of CRs was reported in very few cases, with rebound growth present in 7 out of 12 patients (58.3%); however, the size reached by the mass was smaller than the initial size, and patients remained hemodynamically stable without requiring a new cycle of mTORi.^16^ Ng et al. propose extending treatment until achieving a 50 to 70% reduction in CR size or improvement in obstructive gradient.^26^ Considering the variability in reported data, treatment duration should be individualized and guided by clinical manifestations, hemodynamic alterations, CR size during echocardiographic follow-up, and the potential for rebound growth upon discontinuing mTORi.

Like findings from other studies, most adverse events found in our review were mild and dose-dependent.^18,23,32,33^ Although standardized scales for classifying adverse events are infrequently used in case reports, the majority of events in our review would be categorized as mild (grade 1) or moderate (grade 2), with very few cases classified as severe (grade 3) or potentially life-threatening (grade 4), according to the Common Terminology Criteria for Adverse Events scale.^34^ Dyslipidemia (hypercholesterolemia or hypertriglyceridemia) represents by far the primary reported adverse event, not only by us but also by Sugalska et al. and Cleary et al.^16,24^

Stomatitis has been reported by other authors as the most frequent adverse event in young children receiving mTORi treatment.^32,35,36^ Other commonly reported adverse events include transient neutropenia or lymphopenia, anemia, upper respiratory tract infections, gastrointestinal symptoms (diarrhea or vomiting), elevated liver enzymes, and infantile acne, mostly classified as grade 1 or 2.^16,24,33,36,37^ Non-infectious pneumonitis, although rare in patients under 3 years old,^34^ can be a severe adverse event (grade 3 or 4).^35^ Most of these adverse events can be managed by reducing the dose of mTORi and less frequently by temporary or permanent discontinuation of treatment.^32,36^

This systematic review stands out for being the most recent and complete review related to the effectiveness and safety of mTORi in the treatment of CRs in the neonatal period; however, publication bias cannot be ruled out considering that most of the information came from observational studies. In some cases, it was not possible to recover the missing information, or it was of low quality.

### Recommendations

Neonates and infants are predisposed to achieving high serum levels of mTORi, so the first serum level measurement of the medication should be done within the first week after initiation.

It is reasonable in newborns and infants to use lower doses of mTORi than those approved for other manifestations associated with TSC, adjusting the dose according to the target serum level. Based on our study, an average daily dose of 1.03 mg/m²/day for everolimus and 1.37 mg/m²/day for sirolimus appears to be effective and safe. The most frequently used target serum level of mTORi was 5-15 ng/mL. These doses should be used carefully, considering that the dose of mTORi in neonates is not yet standardized.

More specific information on the pharmacokinetics of mTORi in neonates is needed to ensure optimal dose selection in future clinical trials.

The duration of treatment should be individualized and guided by clinical manifestations, hemodynamic alterations, and the size of cardiac rhabdomyoma during echocardiographic follow-up, as well as the potential for rebound growth upon discontinuing mTORi.

## Conclusion

The use of mTORi in the treatment of symptomatic CRs in neonates at a lower dose than those approved for other manifestations associated with TSC appears to be effective and safe. The average day dose of mTOR inhibitors found in this review was 1.03 mg/m²/day for everolimus and 1.37 mg/m²/day for sirolimus. The most frequently used target serum level of mTORi was 5-15 ng/mL. In our review, all included cases reported a significant reduction in CR size, hemodynamic improvement, and/or resolution of arrhythmia. The speed of mass reduction is significant within the first month of treatment. Rebound growth is an expected effect and may sometimes require restarting treatment. Most adverse events found in our review were mild and dose-dependent. Randomized controlled clinical trials are required.

## Supplementary information

PRISMA 2020 Checklist

SUPPLEMENTARY MATERIAL

## Data Availability

All data generated or analysed during this study are included in this published article.
